# Supplementary material for: A Likelihood Approach for Real-Time Calibration of Stochastic Compartmental Epidemic Models
Source: PLoS Comput Biol. 2017 Jan 17;13(1):e1005257. doi: 10.1371/journal.pcbi.1005257 (PMC5240920; doi:10.1371/journal.pcbi.1005257)
Supplement: S1 File — (TAR.GZ) [file pcbi.1005257.s014.tar.gz › HSPH_Online-SI-Revision/Additions/MSS11/n100-extreme-SEITR_noisy/n100-extreme-SEITR_noisy_NEW2_table4.pdf]

1	newly 3 weeks spec	1111.04	2503.46	{2434., 2563.}	125.326	0.352722	newly 3 weeks cumul	5404.68	9929.37	{9826., 10040.}	83.718	0.264136	newly 5 weeks spec	368.386	1450.82	{1403., 1504.}	293.831	0.595158	newly 5 weeks cumul	6347.06	13297.1	{13181., 13428.}	109.501	0.321175	newly 46 weeks cumul	9732.42	17825.5	{17711., 17915.}	83.1563	0.262817
2	newly 3 weeks spec	909.	1035.36	{997., 1077.}	13.901	0.0563207	newly 3 weeks cumul	5060.	5604.24	{5517., 5691.}	10.7557	0.0443317	newly 5 weeks spec	300.	366.18	{341., 393.}	22.06	0.0859089	newly 5 weeks cumul	5879.	6589.61	{6483., 6702.}	12.0873	0.0495232	newly 54 weeks cumul	6291.	7129.35	{7016., 7244.}	13.3262	0.054299
3	newly 3 weeks spec	1244.	1509.72	{1458., 1558.}	21.3601	0.08393	newly 3 weeks cumul	6664.	7665.33	{7560., 7779.}	15.026	0.0607686	newly 5 weeks spec	463.	587.16	{556., 625.}	26.8164	0.102773	newly 5 weeks cumul	7872.	9191.54	{9072., 9308.}	16.7624	0.0672829	newly 56 weeks cumul	8546.	10158.6	{10031., 10281.}	18.87	0.0750517
4	newly 3 weeks spec	1162.	1182.62	{1141., 1227.}	2.99139	0.0127784	newly 3 weeks cumul	6474.	6281.85	{6196., 6369.}	2.97853	0.013161	newly 5 weeks spec	412.	426.06	{401., 450.}	4.84466	0.0203866	newly 5 weeks cumul	7613.	7422.59	{7318., 7528.}	2.52187	0.0111173	newly 54 weeks cumul	8186.	8070.06	{7963., 8194.}	1.53213	0.00672111
5	newly 3 weeks spec	334.	373.02	{346., 400.}	11.976	0.0486249	newly 3 weeks cumul	2304.	2485.42	{2421., 2545.}	7.87413	0.0328378	newly 5 weeks spec	78.	99.69	{86., 112.}	27.8846	0.104072	newly 5 weeks cumul	2553.	2777.56	{2708., 2839.}	8.79593	0.0365245	newly 56 weeks cumul	2676.	2880.93	{2796., 2953.}	7.65807	0.0319599
6	newly 3 weeks spec	210.	742.66	{708., 774.}	253.648	0.548297	newly 3 weeks cumul	1688.	4249.93	{4176., 4326.}	151.773	0.400971	newly 5 weeks spec	40.	246.93	{224., 268.}	517.325	0.789507	newly 5 weeks cumul	1825.	4927.02	{4843., 5021.}	169.974	0.43128	newly 55 weeks cumul	1849.	5263.48	{5178., 5369.}	184.666	0.45429
7	newly 3 weeks spec	1990.	2173.06	{2108., 2230.}	9.19899	0.0381005	newly 3 weeks cumul	8888.	8909.15	{8798., 9036.}	0.818519	0.00354644	newly 5 weeks spec	982.	1202.2	{1156., 1247.}	22.4236	0.0876869	newly 5 weeks cumul	11272.	11736.3	{11613., 11867.}	4.11888	0.0175127	newly 47 weeks cumul	13516.	15122.9	{14984., 15276.}	11.8885	0.0487739
8	newly 3 weeks spec	1262.	945.48	{907., 987.}	25.0808	0.125635	newly 3 weeks cumul	6258.	5121.94	{5047., 5214.}	18.1537	0.0870388	newly 5 weeks spec	510.	338.2	{315., 367.}	33.6863	0.179224	newly 5 weeks cumul	7632.	6025.29	{5910., 6130.}	21.0523	0.102696	newly 54 weeks cumul	8594.	6524.56	{6408., 6640.}	24.0801	0.119683
9	newly 3 weeks spec	351.	373.66	{347., 405.}	7.63533	0.0316521	newly 3 weeks cumul	2373.	2478.93	{2419., 2549.}	4.49515	0.0190116	newly 5 weeks spec	96.	96.83	{84., 109.}	8.53125	0.036703	newly 5 weeks cumul	2626.	2765.08	{2687., 2847.}	5.31074	0.0223808	newly 56 weeks cumul	2718.	2870.53	{2794., 2952.}	5.61553	0.0236308
10	newly 3 weeks spec	2417.	1901.18	{1852., 1952.}	21.3413	0.10435	newly 3 weeks cumul	10500.	8864.26	{8769., 8967.}	15.5785	0.0735626	newly 5 weeks spec	1167.	848.24	{810., 884.}	27.3145	0.138826	newly 5 weeks cumul	13414.	10990.9	{10901., 11097.}	18.0641	0.0865377	newly 52 weeks cumul	16196.	12676.3	{12559., 12779.}	21.7317	0.106424
11	newly 3 weeks spec	2013.	1770.03	{1701., 1826.}	12.07	0.0560035	newly 3 weeks cumul	9231.	8313.33	{8208., 8414.}	9.94118	0.0454967	newly 5 weeks spec	1003.	774.24	{739., 811.}	22.8076	0.112786	newly 5 weeks cumul	11702.	10256.5	{10119., 10377.}	12.3529	0.0572835	newly 51 weeks cumul	13920.	11757.2	{11603., 11891.}	15.5375	0.0733573
12	newly 3 weeks spec	1233.	1630.57	{1576., 1684.}	32.2441	0.121232	newly 3 weeks cumul	6317.	7595.35	{7521., 7690.}	20.2367	0.0800201	newly 5 weeks spec	542.	736.33	{695., 776.}	35.8542	0.132727	newly 5 weeks cumul	7681.	9433.82	{9336., 9548.}	22.8202	0.0892558	newly 52 weeks cumul	8581.	10905.9	{10790., 11016.}	27.0935	0.104108
13	newly 3 weeks spec	1573.	1403.51	{1351., 1451.}	10.775	0.0496773	newly 3 weeks cumul	7548.	6889.68	{6808., 6977.}	8.72178	0.039653	newly 5 weeks spec	706.	583.12	{550., 614.}	17.4051	0.083418	newly 5 weeks cumul	9330.	8375.65	{8277., 8457.}	10.2288	0.0468801	newly 53 weeks cumul	10733.	9416.74	{9299., 9529.}	12.2637	0.05684
14	newly 3 weeks spec	2628.	2618.05	{2539., 2683.}	1.50875	0.00658224	newly 3 weeks cumul	11099.	10527.4	{10419., 10661.}	5.15001	0.0229796	newly 5 weeks spec	1313.	1463.21	{1417., 1524.}	11.4402	0.0468548	newly 5 weeks cumul	14282.	13961.1	{13833., 14092.}	2.24674	0.00988053	newly 46 weeks cumul	17443.	18166.5	{18013., 18312.}	4.14757	0.017639
15	newly 3 weeks spec	1421.	2635.33	{2567., 2699.}	85.456	0.268156	newly 3 weeks cumul	6948.	10640.8	{10512., 10742.}	53.1488	0.1851	newly 5 weeks spec	621.	1479.11	{1428., 1524.}	138.182	0.37678	newly 5 weeks cumul	8459.	14103.6	{13958., 14217.}	66.7294	0.222002	newly 47 weeks cumul	9505.	18385.2	{18238., 18512.}	93.427	0.286508
16	newly 3 weeks spec	1685.	2485.69	{2413., 2554.}	47.5187	0.168758	newly 3 weeks cumul	8356.	10385.8	{10266., 10503.}	24.292	0.0944284	newly 5 weeks spec	644.	1314.17	{1266., 1366.}	104.064	0.309585	newly 5 weeks cumul	10017.	13521.5	{13398., 13633.}	34.9858	0.130277	newly 50 weeks cumul	11020.	16957.5	{16809., 17103.}	53.8794	0.187171
17	newly 3 weeks spec	244.	372.04	{325., 403.}	52.4754	0.181876	newly 3 weeks cumul	2213.	2741.31	{2651., 2826.}	23.873	0.0928329	newly 5 weeks spec	41.	84.63	{67., 103.}	106.415	0.309215	newly 5 weeks cumul	2358.	3004.54	{2888., 3105.}	27.419	0.105068	newly 56 weeks cumul	2381.	3083.46	{2952., 3201.}	29.5027	0.112096
18	newly 3 weeks spec	946.	772.29	{732., 809.}	18.3626	0.0884248	newly 3 weeks cumul	4981.	4417.64	{4336., 4486.}	11.3102	0.0521674	newly 5 weeks spec	314.	253.49	{231., 275.}	19.2707	0.0939222	newly 5 weeks cumul	5835.	5111.47	{5025., 5194.}	12.3998	0.0575321	newly 55 weeks cumul	6316.	5450.4	{5347., 5533.}	13.7049	0.0640504
19	newly 3 weeks spec	2246.	2731.45	{2666., 2793.}	21.614	0.0849128	newly 3 weeks cumul	9933.	11680.2	{11576., 11789.}	17.5893	0.0703561	newly 5 weeks spec	1136.	1380.75	{1337., 1436.}	21.5449	0.0845541	newly 5 weeks cumul	12584.	14996.1	{14873., 15120.}	19.1682	0.0761509	newly 50 weeks cumul	14784.	18290.7	{18132., 18479.}	23.7195	0.0924283
20	newly 3 weeks spec	688.	748.73	{717., 784.}	8.85029	0.0365667	newly 3 weeks cumul	3837.	4253.51	{4179., 4342.}	10.8551	0.0447143	newly 5 weeks spec	219.	244.92	{221., 269.}	12.6027	0.0506829	newly 5 weeks cumul	4426.	4928.91	{4849., 5019.}	11.3626	0.0466997	newly 55 weeks cumul	4693.	5266.8	{5170., 5366.}	12.2267	0.0500498
21	newly 3 weeks spec	566.	372.73	{347., 403.}	34.1466	0.182221	newly 3 weeks cumul	3401.	2490.61	{2423., 2557.}	26.7683	0.135382	newly 5 weeks spec	156.	97.4	{84., 111.}	37.5641	0.20723	newly 5 weeks cumul	3828.	2778.93	{2705., 2845.}	27.4052	0.139182	newly 56 weeks cumul	3988.	2881.05	{2802., 2953.}	27.757	0.141299
22	newly 3 weeks spec	1150.	1630.36	{1568., 1687.}	41.7704	0.151406	newly 3 weeks cumul	5834.	7594.59	{7493., 7684.}	30.1781	0.114516	newly 5 weeks spec	400.	736.53	{701., 765.}	84.1325	0.264849	newly 5 weeks cumul	6931.	9423.6	{9323., 9535.}	35.9631	0.133404	newly 52 weeks cumul	7573.	10893.9	{10787., 11034.}	43.8522	0.157901
23	newly 3 weeks spec	361.	482.99	{443., 522.}	33.7922	0.125596	newly 3 weeks cumul	2348.	2848.64	{2768., 2925.}	21.322	0.0838489	newly 5 weeks spec	79.	152.15	{132., 170.}	92.5949	0.28252	newly 5 weeks cumul	2588.	3270.02	{3188., 3375.}	26.3532	0.101488	newly 53 weeks cumul	2670.	3460.07	{3359., 3574.}	29.5906	0.112457
24	newly 3 weeks spec	617.	873.76	{842., 910.}	41.6143	0.150907	newly 3 weeks cumul	3939.	4928.68	{4828., 5044.}	25.1252	0.0972956	newly 5 weeks spec	171.	295.52	{271., 318.}	72.8187	0.23674	newly 5 weeks cumul	4404.	5734.29	{5635., 5840.}	30.2064	0.11459	newly 55 weeks cumul	4602.	6143.19	{6029., 6262.}	33.4896	0.125403
25	newly 3 weeks spec	1330.	1755.99	{1694., 1812.}	32.0293	0.120493	newly 3 weeks cumul	7007.	8279.59	{8162., 8405.}	18.1617	0.0724495	newly 5 weeks spec	443.	779.36	{727., 829.}	75.9278	0.244932	newly 5 weeks cumul	8227.	10234.4	{10069., 10378.}	24.4003	0.0947946	newly 51 weeks cumul	8870.	11740.5	{11582., 11910.}	32.3621	0.121736
26	newly 3 weeks spec	920.	1944.77	{1886., 2003.}	111.388	0.324966	newly 3 weeks cumul	5136.	8449.51	{8337., 8555.}	64.5154	0.216186	newly 5 weeks spec	345.	977.3	{933., 1019.}	183.275	0.45195	newly 5 weeks cumul	6057.	10806.	{10680., 10933.}	78.4052	0.251389	newly 49 weeks cumul	6590.	13105.8	{12976., 13233.}	98.8739	0.298563
27	newly 3 weeks spec	2118.	1947.56	{1894., 2000.}	8.04721	0.0365375	newly 3 weeks cumul	9250.	8450.85	{8369., 8531.}	8.63946	0.0392569	newly 5 weeks spec	1079.	980.2	{941., 1019.}	9.1696	0.0419843	newly 5 weeks cumul	11803.	10812.2	{10695., 10933.}	8.39481	0.0380946	newly 49 weeks cumul	14480.	13116.5	{12989., 13245.}	9.41623	0.0429648
28	newly 3 weeks spec	196.	610.	{571., 646.}	211.224	0.492643	newly 3 weeks cumul	1632.	3683.19	{3606., 3761.}	125.686	0.353435	newly 5 weeks spec	32.	185.02	{166., 210.}	478.188	0.760342	newly 5 weeks cumul	1750.	4205.85	{4110., 4294.}	140.334	0.380749	newly 56 weeks cumul	1781.	4434.97	{4328., 4548.}	149.016	0.396151
29	newly 3 weeks spec	1912.	1872.55	{1816., 1937.}	2.60826	0.0115182	newly 3 weeks cumul	9043.	8861.04	{8774., 8966.}	2.01216	0.00884216	newly 5 weeks spec	849.	816.57	{777., 858.}	4.36867	0.0195656	newly 5 weeks cumul	11162.	10913.8	{10796., 11030.}	2.22335	0.00977902	newly 53 weeks cumul	12786.	12460.8	{12329., 12591.}	2.54333	0.0112037
30	newly 3 weeks spec	1221.	1292.39	{1240., 1340.}	5.87961	0.0246505	newly 3 weeks cumul	6812.	7275.56	{7179., 7364.}	6.80505	0.0285722	newly 5 weeks spec	428.	433.18	{413., 456.}	3.59346	0.0154602	newly 5 weeks cumul	7969.	8453.57	{8353., 8542.}	6.08069	0.0256191	newly 56 weeks cumul	8592.	9040.17	{8923., 9122.}	5.21613	0.0220643
31	newly 3 weeks spec	1586.	2124.38	{2034., 2193.}	33.9458	0.126209	newly 3 weeks cumul	7252.	8757.69	{8532., 8799.}	20.7624	0.0815678	newly 5 weeks spec	689.	1157.3	{1089., 1202.}	67.9681	0.223926	newly 5 weeks cumul	9024.	11487.6	{11191., 11519.}	27.3003	0.104343	newly 46 weeks cumul	10290.	14611.9	{14151., 14562.}	42.0007	0.151359
32	newly 3 weeks spec	2184.	1613.86	{1539., 1680.}	26.1053	0.131673	newly 3 weeks cumul	9583.	7563.75	{7427., 7676.}	21.0712	0.1028	newly 5 weeks spec	1094.	722.85	{656., 766.}	33.926	0.180691	newly 5 weeks cumul	12124.	9368.58	{9072., 9508.}	22.727	0.11203	newly 52 weeks cumul	14626.	10797.3	{10229., 10998.}	26.1772	0.13194
33	newly 3 weeks spec	643.	375.41	{341., 407.}	41.6159	0.234678	newly 3 weeks cumul	3804.	2487.57	{2427., 2548.}	34.6065	0.184544	newly 5 weeks spec	197.	98.34	{83., 113.}	50.0812	0.305017	newly 5 weeks cumul	4382.	2776.24	{2700., 2834.}	36.6445	0.198298	newly 56 weeks cumul	4693.	2879.15	{2797., 2946.}	38.6501	0.212275
34	newly 3 weeks spec	883.	1240.67	{1198., 1280.}	40.5062	0.14755	newly 3 weeks cumul	4731.	5929.91	{5843., 6013.}	25.3416	0.0980687	newly 5 weeks spec	278.	534.39	{503., 570.}	92.2266	0.283325	newly 5 weeks cumul	5501.	7280.85	{7177., 7360.}	32.355	0.12172	newly 52 weeks cumul	5915.	8297.93	{8180., 8397.}	40.2862	0.14699
35	newly 3 weeks spec	239.	371.8	{346., 393.}	55.5649	0.191351	newly 3 weeks cumul	1921.	2480.06	{2418., 2531.}	29.1026	0.110864	newly 5 weeks spec	50.	100.02	{87., 114.}	100.04	0.298325	newly 5 weeks cumul	2064.	2769.04	{2703., 2827.}	34.1589	0.127552	newly 56 weeks cumul	2096.	2874.48	{2803., 2935.}	37.1412	0.137093
36	newly 3 weeks spec	2432.	1865.8	{1812., 1918.}	23.2813	0.115222	newly 3 weeks cumul	10891.	8965.79	{8876., 9058.}	17.6771	0.0844942	newly 5 weeks spec	1211.	792.17	{755., 832.}	34.5855	0.184649	newly 5 weeks cumul	13782.	10971.4	{10872., 11076.}	20.3936	0.0990657	newly 52 weeks cumul	16479.	12405.3	{12271., 12547.}	24.7205	0.123339
37	newly 3 weeks spec	1023.	1405.61	{1352., 1458.}	37.4008	0.137815	newly 3 weeks cumul	5532.	6890.78	{6808., 6971.}	24.5622	0.095365	newly 5 weeks spec	383.	583.57	{558., 614.}	52.3681	0.182557	newly 5 weeks cumul	6497.	8378.81	{8282., 8454.}	28.9643	0.110453	newly 53 weeks cumul	7036.	9419.96	{9320., 9543.}	33.8823	0.126704
38	newly 3 weeks spec	915.	1414.87	{1372., 1455.}	54.6306	0.189139	newly 3 weeks cumul	5124.	6598.45	{6511., 6696.}	28.7754	0.109808	newly 5 weeks spec	323.	631.92	{597., 671.}	95.6409	0.291058	newly 5 weeks cumul	5962.	8178.43	{8075., 8301.}	37.1759	0.137255	newly 50 weeks cumul	6391.	9426.75	{9284., 9566.}	47.5004	0.168766
39	newly 3 weeks spec	1339.	1533.14	{1481., 1587.}	14.4989	0.0586393	newly 3 weeks cumul	6703.	7431.18	{7339., 7530.}	10.8635	0.0447645	newly 5 weeks spec	464.	637.72	{600., 673.}	37.4397	0.137632	newly 5 weeks cumul	7906.	9054.4	{8953., 9164.}	14.5257	0.0588826	newly 52 weeks cumul	8666.	10198.7	{10071., 10353.}	17.6862	0.0707047
40	newly 3 weeks spec	1822.	1426.02	{1385., 1465.}	21.7333	0.106539	newly 3 weeks cumul	8114.	6743.38	{6648., 6822.}	16.892	0.0803814	newly 5 weeks spec	874.	625.28	{590., 659.}	28.4577	0.145905	newly 5 weeks cumul	10273.	8308.25	{8194., 8401.}	19.1254	0.0922076	newly 53 weeks cumul	12291.	9508.85	{9378., 9627.}	22.6357	0.111482
41	newly 3 weeks spec	362.	373.25	{334., 409.}	7.03591	0.0298705	newly 3 weeks cumul	2774.	2747.84	{2640., 2846.}	2.29488	0.0100684	newly 5 weeks spec	74.	86.89	{69., 102.}	19.9324	0.0766401	newly 5 weeks cumul	2996.	3014.47	{2890., 3125.}	2.50901	0.0108405	newly 56 weeks cumul	3059.	3094.39	{2968., 3230.}	2.75221	0.0118277
42	newly 3 weeks spec	2612.	2636.28	{2563., 2707.}	1.74119	0.00749717	newly 3 weeks cumul	10679.	10620.1	{10513., 10721.}	0.754284	0.00329204	newly 5 weeks spec	1374.	1480.17	{1429., 1535.}	7.72707	0.0321472	newly 5 weeks cumul	13825.	14079.4	{13969., 14209.}	1.84043	0.00791034	newly 47 weeks cumul	17141.	18379.6	{18232., 18552.}	7.22595	0.0302908
43	newly 3 weeks spec	2253.	1893.19	{1828., 1938.}	15.9703	0.0756682	newly 3 weeks cumul	10165.	8850.09	{8735., 8958.}	12.9357	0.0601791	newly 5 weeks spec	1018.	847.01	{802., 890.}	16.7967	0.0801883	newly 5 weeks cumul	12779.	10963.5	{10862., 11056.}	14.2071	0.066561	newly 52 weeks cumul	15131.	12636.5	{12522., 12745.}	16.486	0.0782524
44	newly 3 weeks spec	1384.	1537.57	{1487., 1588.}	11.0961	0.0455705	newly 3 weeks cumul	6755.	7436.63	{7322., 7525.}	10.0907	0.0417277	newly 5 weeks spec	626.	636.51	{591., 676.}	4.22204	0.0180086	newly 5 weeks cumul	8295.	9061.45	{8933., 9167.}	9.2399	0.0383607	newly 52 weeks cumul	9359.	10206.6	{10061., 10337.}	9.05642	0.0376274
45	newly 3 weeks spec	1196.	941.89	{899., 985.}	21.2467	0.103973	newly 3 weeks cumul	6409.	5109.43	{5017., 5194.}	20.2773	0.0984551	newly 5 weeks spec	536.	342.38	{318., 366.}	36.1231	0.195433	newly 5 weeks cumul	7757.	6016.31	{5904., 6102.}	22.4402	0.110405	newly 54 weeks cumul	8640.	6510.54	{6378., 6640.}	24.6465	0.122943
46	newly 3 weeks spec	1149.	373.56	{332., 413.}	67.4883	0.489114	newly 3 weeks cumul	6165.	2744.37	{2650., 2854.}	55.4847	0.351699	newly 5 weeks spec	377.	85.32	{68., 104.}	77.3687	0.651076	newly 5 weeks cumul	7203.	3009.9	{2909., 3146.}	58.2132	0.379183	newly 56 weeks cumul	7768.	3089.28	{2980., 3219.}	60.2307	0.400677
47	newly 3 weeks spec	1358.	1760.55	{1703., 1821.}	29.6429	0.112626	newly 3 weeks cumul	7212.	8294.48	{8182., 8408.}	15.0094	0.0607094	newly 5 weeks spec	550.	773.85	{729., 818.}	40.7	0.14789	newly 5 weeks cumul	8640.	10238.7	{10095., 10390.}	18.5034	0.0737057	newly 51 weeks cumul	9536.	11730.3	{11556., 11885.}	23.0106	0.089918
48	newly 3 weeks spec	874.	1176.54	{1126., 1224.}	34.6156	0.128848	newly 3 weeks cumul	5250.	6268.49	{6177., 6351.}	19.3998	0.076981	newly 5 weeks spec	248.	425.59	{396., 458.}	71.6089	0.23392	newly 5 weeks cumul	5990.	7409.93	{7322., 7486.}	23.705	0.0923664	newly 54 weeks cumul	6323.	8057.99	{7977., 8155.}	27.4393	0.105281
49	newly 3 weeks spec	879.	1235.47	{1191., 1290.}	40.554	0.147635	newly 3 weeks cumul	4780.	5914.77	{5819., 5988.}	23.74	0.0924799	newly 5 weeks spec	321.	537.49	{510., 569.}	67.4424	0.223392	newly 5 weeks cumul	5641.	7265.6	{7177., 7374.}	28.7999	0.109893	newly 52 weeks cumul	6148.	8281.99	{8162., 8400.}	34.7103	0.129376
50	newly 3 weeks spec	1513.	1401.31	{1350., 1447.}	7.38202	0.0334544	newly 3 weeks cumul	7746.	6884.89	{6789., 6960.}	11.1168	0.0511976	newly 5 weeks spec	636.	581.84	{550., 616.}	8.58491	0.0393551	newly 5 weeks cumul	9438.	8367.23	{8271., 8457.}	11.3453	0.0523148	newly 53 weeks cumul	10556.	9405.62	{9313., 9522.}	10.8979	0.050127
